# Bibliography

**Published:** 1902-04

**Authors:** 


### Bibliography.

The Röntgen Rays in Medicine and Surgery, as an Aid in Diagnosis and as a Therapeutic Agent. Designed for the Use of Practitioners and Students. By Francis H. Williams, M.D. (Harv.), Graduate of the Massachusetts Institute of Technology; Visiting Physician at the Boston City Hospital; Fellow of the Massachusetts Medical Society, etc. With three hundred and ninety-one illustrations. The Macmillan Company, New York; Macmillan & Co., London, 1901.

It is just seven years since Röntgen announced his discovery of the X-rays. The interest that this announcement excited was only equalled by the enthusiasm with which the matter was taken up by physicists throughout the civilized world. It opened an entirely new avenue by which disease in some forms could be diagnosticated. Its full value in this direction was not then understood, and the time is yet too short to fully realize all the possibilities connected with it and which will be without doubt elucidated by further study. In fact, the author clearly states that, "The following pages are rather a report of progress than a final presentation of this growing subject."

This is altogether too modest a statement of this splendid production of six hundred and thirty-eight pages. When the X-rays were first investigated it was supposed that their use would be confined entirely to the hard structures of the human organism, locating bullets, etc., but although so short a time has elapsed since the discovery of these rays, the author describes advances in their use in diagnosticating disease almost bewildering to one not wholly familiar with the progress made. To the reviewer this is something near akin to a revelation, and he feels that the medical profession in all its branches owes the author a special feeling of gratitude for thus placing before it that which amounts to new discoveries in the science of disease.

The author thus expresses himself in regard to a highly honored correspondent of this journal, whose work in the direction of the Röntgen rays has not been surpassed by any other. He says, "I wish it were possible for me to express the gratitude I feel towards Dr. William Rollins for his unfailing aid in my efforts to obtain apparatus suitable for these examinations, and particularly for the new and better forms of vacuum tubes that he has devised."

Chapter I. is devoted to the "Nature and Properties of the X-Rays." This is a very clear statement, and is followed by "X-Ray Equipment," a very important chapter for those anticipating experimental work in this direction. All the new forms of apparatus are fully illustrated, including those instruments introduced by Dr. Rollins.

Chapter III., on "Methods for making X-Ray Examinations with the Fluorescent Screen and X-Ray Photograph," is exceedingly interesting, and is so thoroughly illustrated that there is no difficulty in reaching at least a moderate degree of comprehension of the author's methods. This chapter must be studied in detail to properly understand it, but it seems to the reviewer to be the foundation of all the work which follows. The knowledge must be, however, part of laboratory training and cannot be acquired by mere reading. In fact, the entire book is so thoroughly practical in its details that its proper place is in the pathological workshop, rather than in the library. This, however, only increases its value as an original production.

Chapter V., on "Pulmonary Tuberculosis," will be read with special interest, as the idea that anything satisfactory could be developed in this disease by X-rays is a comparatively new thought, and to which little credence has been given. The author says, "It was early recognized by many practitioners in various countries that the dense lung in this disease would cast a shadow which might be observed on the fluorescent screen. It seemed to me, also, that we might find in the X-rays another means of recognizing pulmonary tuberculosis in its earliest stage, and with this end in view I took every opportunity to examine early cases of this disease." The author then follows with "Appearances seen on the Fluorescent Screen," in this disease fully illustrated. He qualifies the value of the work further along in the chapter, for he says, "The diagnosis of phthisis is not made by the X-ray examinations alone, but it does give us early warning of a departure from the normal in the lung, which puts us on our guard and enables us, in conjunction with the history, etc., to make the diagnosis, and thus in many early cases of tuberculosis to arrest its progress by proper care."

Of pneumonia the author says, "A pneumonia in its early stages, or even through its whole course, may give no signs by auscultation and percussion, and the physician may find it difficult to make the diagnosis. In some of these cases a doubtful diagnosis may be made a more certain one by the use of the X-rays." Three cases are cited as illustration of this.

It is not possible to follow the author chapter by chapter, but each has an interest of its own. The X-ray is not equal to showing all malformations distinctly, but in connection with other well-understood methods of diagnosis may be made of exceeding value. It is quite evident that the future will continue to develop new methods of observation, enabling the observer to differentiate more clearly pathogenic conditions. The author says, "New growths in the abdomen are not easily recognized by an X-Ray examination. We may get some suggestion of their presence if they affect the outline of the diaphragm; or, if the growth is well marked and dense, it may cast a shadow on the fluorescent screen or be seen in the negative."

In Chapter XVI. the author treats of the "Therapeutic Uses of the X-Rays," and says of this, "The accounts given in the medical journals of the therapeutic uses of the X-rays in diseases of the skin have seemed to many practitioners beyond belief." He then proceeds to cite cases that go far to demonstrate its value as a therapeutic agent in this direction. He says of the X-rays in cancer that, "This new therapeutic agent gives us a method of treatment which is painless and useful in certain, if not all, forms of external cancer of not great depth; how efficient it will prove to be it will take two or three years to decide, but if there is a recurrence, treatment could be again instituted."

The author quotes two cases where the X-rays proved to have analgesic properties, one where a youth had been shot in the thigh. A long exposure to the X-rays produced entire freedom from pain. A patient suffering from gall-stones had a similar experience.

"Fractures and Dislocations," as a matter of course, find an extended and important place in the volume. These are very fully and clearly illustrated. "Foreign Bodies" naturally follow, and the clearness of some of the illustrations are in the direction of a revelation to some who have been accustomed to obscure definition. In one on page 545 a needle in the hand is so perfectly delineated that the eye is as distinct as though lying in the open hand.

"Diseases of the Bones and of the Joints" are appropriately given much space.

Chapter XXIII. is given up to "Dental Surgery." The author says here that, "For the successful use of the X-rays in dentistry, sharp definition in negatives is necessary, and differentiation is required between tissues that do not differ very much in the obstruction they offer to the passage of the X-rays. The roots of the teeth are only a little less permeable to the rays than the surrounding bone, and therefore it is difficult to get a clear picture of their ends." Then follows a description of suitable apparatus for this work.

The importance of the X-rays in dentistry is only just beginning to be appreciated, notwithstanding the earnest efforts of Dr. Rollins and Clapp, of Boston, and Dr. W. A. Price, of Cleveland. One reason is that mentioned by Dr. Williams,—that the difficulty of securing a clear picture is in a measure confusing. This may be overcome, and it would seem proper to make this study a part of the curriculum of all well-regulated dental colleges, for until this is done it is hopeless to expect the X-ray to become of value in the diagnoses of certain diseases of the oral cavity.

The examination of calculi and an Appendix constitute the closing chapters of this interesting and exceedingly valuable book. It should be in the library of every professional man, for while the work of examination by the X-rays must necessarily fall into the hands of specialists, the general practitioner is required to become familiar with its possibilities and its limitations in order to meet the growing demand for its use. No other work with which the reviewer is acquainted meets the demand as fully as this volume.

The publishers have given the readers a very perfectly prepared book, both in text and illustrations.

QUIZ COMPEND ON IRREGULARITIES OF THE TEETH. By Eugene S. Talbot, M.D., D.D.S., Professor of Dental and Oral Surgery, Northwestern University, Women's Medical School. First Edition. E. M. Clay & Co., Chicago, 1901.

This is a decidedly new departure from the pen of this prolific writer, and while it is called a quiz compend, it cannot be regarded in the same category with the usual compends that flood the market, as it comprises something more than a series of questions and answers, ordinarily very brief and failing to convey a proper comprehension of the subject. Dr. Talbot has endeavored to convey in a very few words his ideas, and he accomplishes this in a way that at once commands the attention of the reader and must claim the interest of the student. It is not often that a book of this description is readable, but this will be found to chain the attention from the beginning to the end.

It naturally follows very much the trend of thought to be found in the author's work on Irregularities, being a condensed statement of his own views and also of the literature of the subject.

The author states in his preface that "This work is not intended to take the place of the larger and more complete works, but to be used as a primer and reference for advanced students. . . . The author has intentionally avoided a discussion of special treatment, since every teacher has, of necessity, his own methods of operation."

This may interfere with its adoption in colleges, but will make it all the more useful as a reference for advanced students and practitioners who desire to become familiarized with facts that necessarily lead up to irregularities. It will no doubt be an incentive to many to follow in the same line of investigation that has made the author's name so prominent in scientific circles.

The four opening chapters are devoted to History, Heredity, Congenital Factors and Maternal Impressions, Post-natal Skull and Jaw Development, and Periods of Stress. Chapter V. begins with "Development of the Cranium and Face."

In Chapter VI., on "Development of the Jaws," exception must be made to the author's statement in the following questions and answers:

Question: Are teeth more liable to decay upon the upper jaw than the lower? Answer: Yes.

Question: Why? Answer: Because the upper is degenerating faster than the lower.

Question: Is degeneracy (or arrest of development) of the jaws and teeth a cause of decay? Answer: Yes. It is a principal cause."

In the reviewer's experience it is not true that the upper jaw is more liable to decay than the lower, except in special instances. The exceptions have nothing to do with degeneracy, if that much abused word is understood. In proportion as teeth are removed from the salivary ducts will caries be more pronounced. Hence the superior molars are less affected than the inferior, and the lower incisors far less than the upper incisors. Tables of extracted teeth are unsafe guides upon which to formulate a theory of degeneration.

It is not possible to agree with all the author's conclusions, but his work on Irregularities has been so recently reviewed that space will not permit critical notices of these disagreements.

As a condensed statement of the author's views it can be warmly recommended, and it will be an aid to a more extended study of his recent work,—in fact, might well go as a companion to it.

